# Magnitude of portal pressure reduction and clinical outcomes in randomized trials

**DOI:** 10.1016/j.jhepr.2026.102028

**Published:** 2026-09-21

**Authors:** Matthew Tam, Irene Pascual, Jarell Jie-Rae Tan, Daniel Veldhuijzen van Zanten, Beverley Kok, Shuen Sung, Janice Y. Kung, Yu Jun Wong, Juan G. Abraldes

**Affiliations:** 1 Liver Unit, Division of Gastroenterology, Department of Medicine, University of Alberta, Edmonton, Canada; 2 Yong Loo Lin School of Medicine, National University of Singapore, Department of Gastroenterology and Hepatology, Changi General Hospital, Singapore, Duke-NUS Medical School, Singapore

**Keywords:** Portal hypertension, Hepatic venous pressure gradient, Meta-regression, Surrogate endpoint, Beta-blockers, Cirrhosis

## Abstract

**Background & Aims:**

Portal hypertension is a major driver of complications of cirrhosis. Observational studies suggest that reductions in hepatic venous pressure gradient (HVPG) improve prognosis, but the magnitude of risk reduction per unit HVPG decrease has not been quantified across randomized controlled trials (RCTs). We performed a meta-regression of RCTs to quantify this relationship.

**Methods:**

We systematically searched for RCTs testing pharmacological interventions against placebo or no treatment in portal hypertension that reported both HVPG measurements and clinical outcomes. The predictor was the between-group difference in HVPG change (treatment minus control, in mmHg). Primary endpoints were all-cause mortality and variceal events, defined hierarchically as variceal progression (when reported) or variceal bleeding. We used random-effects meta-regression, weighted by the inverse variance of the log odds ratio. Sensitivity analyses tested alternative weighting schemes accounting for HVPG measurement error.

**Results:**

Twenty-two RCTs (2,871 patients; 1,991 with HVPG measurements) were included. Each 1 mmHg greater HVPG reduction in the treatment *vs*. control group was associated with significantly lower all-cause mortality (odds ratio [OR] 0.850, 95% CI 0.771–0.937; *p* = 0.004; *I*^2^ = 0%) and fewer variceal events (OR 0.852, 95% CI 0.744–0.976; *p* = 0.032; *I*^2^ = 18.8%). Results were consistent across outcome-based, joint, and iteratively reweighted meta-regression models. Exploratory non-linear analyses showed no evidence against a linear relationship within the observed range.

**Conclusions:**

Each mmHg of HVPG reduction achieved by pharmacological therapy is associated with, at the trial level, ∼15% lower odds of mortality and variceal events. These findings provide a quantitative framework for interpreting HVPG changes in phase II trials and informing the design of phase III studies.

**Impact and implications:**

Lowering portal pressure is central to preventing complications of cirrhosis, but the clinical benefit associated with a given treatment-related reduction in hepatic venous pressure gradient has not been quantified across randomized trials. In 22 trials, each additional 1 mmHg reduction was associated at the trial level with ∼15% lower odds of death and variceal events. This quantitative relationship may help researchers and drug developers interpret phase II hemodynamic results and inform go/no-go decisions and phase III trial design. Because the analysis uses aggregate trial-level data, it does not establish causality or predict benefit for individual patients.

## Highlights

• Meta-regression included 22 RCTs and 2,871 patients with portal hypertension.
• Each 1 mmHg greater HVPG reduction was linked to 15% lower odds of death.
• Each 1 mmHg greater HVPG reduction was linked to fewer variceal events.
• Associations were robust across weighting and measurement-error analyses.
• HVPG effects may guide phase II go/no-go decisions and phase III trial design.

## Graphical abstract

**Figure:**
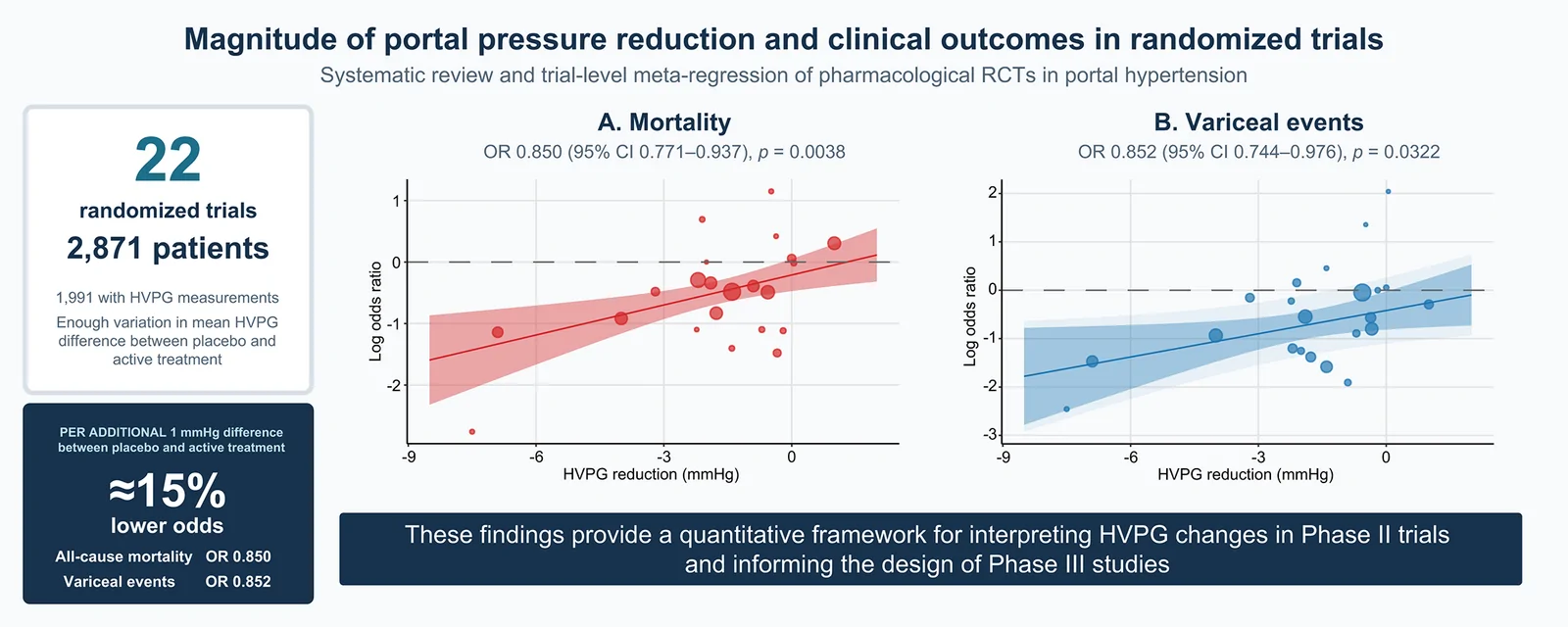

## Introduction

Portal hypertension, defined by a hepatic venous pressure gradient (HVPG) exceeding 5 mmHg, is a major driver of morbidity and mortality in cirrhosis.^1^ The prognostic significance of portal pressure is well established: patients with HVPG values >10 mmHg (clinically significant portal hypertension) face substantially increased risks of decompensation and death.^2^

Observational cohort studies have consistently demonstrated that patients achieving greater HVPG reductions during treatment experience better clinical outcomes.^3^^,^^4^ Patients whose HVPG decreased by more than 10–20% from baseline, or to absolute values <12 mmHg, showed marked reductions in all complications of cirrhosis and improved survival compared with non-responders.^4^ However, these analyses are inherently vulnerable to potential bias. Patients with more favorable underlying pathophysiology may both respond better to treatment and have superior baseline prognosis.^5^ In addition, those experiencing early clinical events may be unable to undergo repeat HVPG measurement, potentially introducing immortal time bias. Most critically, without a control group, observational designs cannot distinguish whether improved outcomes result from HVPG reduction itself or from unmeasured prognostic factors.^6^

Randomized controlled trials (RCTs) provide the methodological framework to address these limitations.^6^ Through randomization, RCTs create groups comparable in prognosis. The between-group comparison, contrasting HVPG reduction in the treatment arm *vs*. the control arm, and relating this difference to the between-group difference in clinical outcomes quantifies the trial-level association between the treatment effect on HVPG and the treatment effect on outcomes, evaluating HVPG reduction as a trial-level surrogate.^6^ Meta-regression across multiple trials with enough variation on treatment effects can then quantify this trial-level surrogate relationship.^7^^,^^8^

This question has direct implications for drug development and clinical practice. HVPG is frequently measured in early-phase trials to establish proof of concept that a drug reduces portal pressure, while phase III trials rely on clinical endpoints.^9^ If a quantitative relationship between HVPG reduction and clinical benefit can be established, it would enable more accurate prediction of phase III outcomes from phase II hemodynamic data and provide a scientific basis for sample size calculations.^10^

Despite the availability of numerous RCTs testing pharmacological interventions in portal hypertension with concurrent HVPG and clinical outcome measurement, no prior analysis has systematically quantified this relationship using meta-regression methodology. We therefore conducted a meta-regression analysis of RCTs examining two primary clinical endpoints: all-cause mortality and variceal events, and an exploratory analysis of variceal bleeding.

## Materials and methods

### Study design and registration

The reporting of this systematic review and meta-regression analysis was guided by the standards of the Preferred Reporting Items for Systematic Review and Meta-Analysis (PRISMA) 2020 guidelines. The review protocol, including eligibility criteria, outcomes, and the analysis plan, was defined before data extraction but was not prospectively registered, and no public protocol was posted. To mitigate selection and reporting bias we used two independent reviewers for screening and extraction with third-reviewer adjudication and prespecified the primary outcomes and the meta-regression model.

### Literature search and study selection

We conducted an initial systematic literature search on July 19, 2020 in Ovid MEDLINE, Ovid Embase, Scopus, and the Cochrane Central Register of Controlled Trials (via Wiley Cochrane Library) to identify all RCTs testing pharmacological interventions for portal hypertension that reported both HVPG measurements and clinical outcomes. The search was subsequently updated using the same search strategies, with the final update conducted in December 2025. The complete search strategy, including search terms and controlled vocabulary, is provided in the Supplementary Materials. Results were imported into Covidence, where two independent reviewers screened titles and abstracts, with full-text assessment of potentially eligible studies. Disagreements were resolved by consensus or adjudication by a third reviewer.

Studies were eligible if they were RCTs comparing active pharmacological treatment *vs*. placebo or no active therapy in adults with cirrhosis and portal hypertension, reported baseline and follow-up HVPG measurements in both groups, and reported at least one clinical outcome of interest (variceal bleeding, all-cause mortality, or variceal development/progression). Studies comparing two active treatments without a control group, those reporting only baseline HVPG, and duplicate publications were excluded.

### Data extraction

For each trial, we extracted study characteristics (design, sample size, follow-up), population characteristics (etiology, Child–Pugh class, prophylaxis context), intervention details (drug, dose, duration), HVPG measurements (baseline, follow-up, and change from baseline with standard deviations in both groups), and clinical outcomes (events in each group). Data were extracted independently by two reviewers, with discrepancies resolved by consensus.

### Handling of missing variance data

Many trials reported HVPG means without variance measures for changes from baseline. When change SDs were not directly reported, we calculated them from standard errors, confidence intervals, or from baseline and follow-up SDs using the formula: SDchange = √(SDbaseline^2^ + SDfollowup^2^ − 2ρ × SDbaseline × SDfollow-up).^11^^,^^12^ We estimated the correlation ρ = 0.687 (median) from five studies reporting sufficient data to back-calculate it empirically. For one study reporting only percentage changes,^13^ we converted to absolute values using baseline means and imputed the SD from the median of other studies. Sensitivity analyses varied ρ from 0.5 to 0.8. For multi-arm trials,[14], [15], [16] we combined treatment arms using standard Cochrane methods to avoid unit-of-analysis errors.

### Outcome definitions

We analyzed two primary outcomes. All-cause mortality was defined as deaths from any cause during trial follow-up. Variceal events were defined hierarchically: variceal development or progression when reported, otherwise variceal bleeding. We combined these into a hierarchical variceal events endpoint for three reasons. First, it maximized the number of trials contributing to the analysis. Second, variceal progression keeps repeat HVPG measurement feasible, whereas variceal bleeding typically precludes it. Third, variceal progression frequently triggers rescue therapy (*e.g.* band ligation), which disconnects subsequent bleeding from the randomized intervention.^17^ As an exploratory outcome, we analyzed variceal bleeding alone.

### Assessment of study quality

Risk of bias was assessed independently by two reviewers using the Cochrane Risk of Bias 2 tool for randomized trials.^12^ Any discrepancy in the study quality assessment was discussed amongst members of the research group and was independently verified by the principal investigator (Table S1).

### Statistical analysis

We conducted random-effects meta-regression to examine the relationship between HVPG reduction and clinical outcomes. For each trial, we calculated the HVPG change difference (treatment change minus control change; negative values indicate greater reduction in the treatment group) and the log odds ratio (OR) for each clinical endpoint. For the regression models this predictor was sign-reversed, so that a positive value denotes the number of mmHg by which HVPG fell further in the treatment arm than in the control arm; all regression coefficients and ORs are therefore expressed per 1 mmHg of greater HVPG reduction in the treatment arm. We then regressed log ORs against HVPG change differences, using restricted maximum likelihood estimation and the Knapp–Hartung adjustment for standard errors. Studies were weighted by the inverse variance of the log OR (primary analysis). Heterogeneity was assessed using Cochran’s Q test, *I*^2^, and τ^2^.

### Sensitivity analyses

We performed sensitivity analyses using different weighting schemes: predictor-based weighting (1/Var[HVPG change]), joint weighting accounting for uncertainty in both outcome and predictor and iteratively reweighted meta-regression (updating joint weights until convergence).^8^^,^^18^^,^^19^ We assessed the impact of correlation assumptions (ρ = 0.5–0.8), performed leave-one-out analyses, and tested a non-linear model (quadratic). To handle trials with zero-event arms without a continuity correction, we additionally fitted a one-stage random-effects logistic (binomial-normal) meta-regression to the event counts directly.^20^ Further, to quantify the attenuation induced by measurement error in the predictor, we applied a SIMEX correction^21^ to the slope using each trial own sampling variance of the between-group HVPG change, with a study-level bootstrap for the corrected confidence interval, and repeated the correction using the test–retest standard deviation of HVPG change reported in our previous study,^10^ converted for each trial into an error variance for the between-group mean difference as SD^2^ × (1/n_treatment + 1/n_control), where n is the number of patients with paired HVPG measurements in each arm. The influence of individual trials was assessed using Cook's distance, DFBETAS, studentized residuals, hat values and covariance ratios.^22^ Predicted trial-level treatment effects at specified HVPG reductions were obtained from the fitted models with their 95% CIs and prediction intervals. All analyses were conducted in R (version 4.4, R Foundation for Statistical Computing, Vienna, Austria) using the *metafor*^23^ package.

## Results

### Study selection and characteristics

The systematic search identified 4,217 records. After removal of 1,450 duplicate records, 2,767 records underwent title and abstract screening, of which 125 underwent full-text review. Twenty-two RCTs met inclusion criteria. One study^14^ had three active treatment arms which were combined, and another^16^ had two active arms also combined using Cochrane methods. After combining multi-arm treatment groups, 22 independent comparisons were available for analysis. A detailed PRISMA flow diagram is provided in Fig. S1.

Table 1 summarizes the characteristics of the included trials. The 22 RCTs enrolled a total of 2,871 patients, of whom 1,991 underwent HVPG measurement. Individual study sample sizes ranged from 23 to 263 patients (mean 130). Non-selective beta-blockers were the most frequently tested drug class (propranolol, carvedilol, nadolol, or timolol in 11 trials),^24^ followed by statins (3 trials), and other agents including spironolactone, midodrine, emricasan, belapectin, simtuzumab, olmesartan, octreotide, and probiotics. Ten trials were conducted in the primary prophylaxis setting, two in secondary prophylaxis, one in prevention of decompensation, and the remainder did not specify or enrolled mixed populations. The median HVPG measurement interval was 6 months (range 0.3–24). The mean HVPG reduction in treatment groups was 2.72 ± 1.88 mmHg compared with 0.95 ± 1.88 mmHg in control groups, yielding a mean overall between-group difference of 1.78 ± 2.10 mmHg. The between-group HVPG change differences ranged from -7.5 to +1.0 mmHg across trials (Fig. 1), showing that the trials provided enough variability in HVPG responses to conduct the meta-regression.

**Table 1:** Characteristics of included studies.

Study	Country	Context	Comparison	Drug target	N (total)	N (HVPG)	HVPG Time (mo)	FU (mo)	Baseline HVPG Ctrl (mmHg)	Baseline HVPG Trt (mmHg)	ΔHVPG Diff (mmHg)	Mortality n (%) C/T	Variceal event n (%) C/T	Bleeding n (%) C/T
Burroughs 1983^24^	UK	Secondary prophylaxis	Propranolol *vs.* placebo	Hepatic inflow	48	19	1	12	21.1 ± 7.9	17.7 ± 4.9	-3.20	5 (23)/4 (15)	11 (50)/12 (46)	11 (50)/12 (46)
Villeneuve 1986^25^ Hemodynamic data in Pomier-Layrargues *et al.*, 1987^26^	Canada	Secondary prophylaxis	Propranolol *vs.* placebo	Hepatic inflow	79	19	0.3	12	21.1 ± 6.2	21.1 ± 6.5	1.00	14 (38)/19 (45)	30 (81)/32 (76)	30 (81)/32 (76)
Conn 1991^27^ Hemodynamic data in Groszmann 1990^28^	USA	Primary prophylaxis	Propranolol *vs.* placebo	Hepatic inflow	102	84	3	3	19.1 ± 5.1	18.2 ± 4.2	-0.90	11 (22)/8 (16)	11 (22)/2 (4)	11 (22)/2 (4)
Jenkins 1997^29^	England	Secondary prophylaxis	Injection sclerotherapy + octreotide *vs.* injection sclerotherapy	Hepatic inflow	32	32	6	6	22.3 ± 2.6	25.4 ± 3.9	-7.50	5 (31)/0 (0)	7 (44)/1 (6)	7 (44)/1 (6)
Abecasis 2003^30^	Argentina	Primary prophylaxis	Nadolol + spironolactone *vs.* nadolol + placebo	Hepatic inflow	100	24	2	22	16.3 ± 3.7	17.7 ± 2.1	-0.70	3 (6)/1 (2)	7 (14)/3 (6)	7 (14)/3 (6)
Merkel 2004^31^	Italy	Primary prophylaxis	Nadolol *vs.* placebo	Hepatic inflow	161	19	24	36	12.3 ± 1.3	12.2 ± 1.1	-1.40	31 (40)/24 (29)	29 (37)/9 (11)	9 (12)/2 (2)
Groszmann 2005^32^	USA	Primary prophylaxis	Timolol *vs.* placebo	Hepatic inflow	213	154	12	54.9	11.7 ± 4.1	11.7 ± 4.3	-0.56	15 (14)/10 (9)	42 (40)/42 (39)	3 (3)/2 (2)
Hidaka 2011^33^	Japan	Primary prophylaxis	Olmesartan *vs.* placebo	Hepatic inflow	48	45	12	48	16.0 ± 2.3	15.9 ± 3.4	-2.00	0 (0)/0 (0)	8 (33)/3 (13)	0 (0)/0 (0)
Gupta 2013^16^	India	Primary prophylaxis	Propranolol + VSL#3 *vs.* propranolol	Adjunctive (microbiome-targeted)	94	75	2	2	19.0 ± 3.2	19.1 ± 4.3	-1.40	2 (6)/1 (2)	1 (3)/3 (5)	1 (3)/3 (5)
Sarin 2013^34^	India	Primary prophylaxis	Propranolol *vs.* placebo	Hepatic inflow	49	49	12	25	14.5 ± 4.8	15.5 ± 5.8	-2.10	1 (4)/2 (8)	6 (25)/7 (28)	1 (4)/2 (8)
Bhardwaj 2016^35^	India	Primary prophylaxis	Carvedilol *vs.* placebo	Hepatic inflow	140	100	12	24	14.7 ± 4.8	14.5 ± 4.3	-0.34	8 (11)/2 (3)	25 (36)/14 (20)	0 (0)/0 (0)
Bishnu 2018^36^	India	Primary prophylaxis	Propranolol + atorvastatin *vs.* propranolol	Hepatic resistance	23	23	1	12	17.2 ± 6.1	15.6 ± 5.3	-2.23	1 (8)/0 (0)	5 (42)/4 (36)	5 (42)/4 (36)
Harrison 2018^15^	USA	Treatment of fibrosis	Simtuzumab *vs.* placebo	Hepatic resistance	258	258	24	96	12.0 ± 4.8	12.3 ± 5.8	0.05	1 (1)/2 (1)	0 (0)/7 (4)	0 (0)/7 (4)
Kumar 2019^13^	India	ACLF	Carvedilol *vs.* placebo	Hepatic inflow	136	91	3	3	18.0 ± 4.2	19.0	-2.20	21 (30)/16 (24)	15 (21)/5 (8)	5 (7)/5 (8)
Villanueva 2019^17^	Spain	Prevention of decompensation	Carvedilol/propranolol *vs.* placebo	Hepatic inflow	201	156	12	36	14.8 ± 3.5	14.5 ± 3.5	-1.90	11 (11)/8 (8)	25 (25)/16 (16)	3 (3)/4 (4)
Chalasani 2019^14^	USA	Primary prophylaxis	Belapectin *vs.* placebo	Hepatic resistance	162	151	12	12	11.6 ± 4.0	12.6 ± 4.2	-0.36	0 (0)/1 (1)	9 (17)/11 (10)	0 (0)/4 (4)
Vijayaraghavan 2020^37^	India	Primary prophylaxis	Carvedilol + simvastatin *vs.* carvedilol	Hepatic resistance	220	121	3	3	17.2 ± 3.2	17.7 ± 3.4	-0.20	3 (3)/1 (1)	2 (2)/2 (2)	2 (2)/2 (2)
Garcia-Tsao 2020^38^	International	Primary prophylaxis	Emricasan *vs.* placebo	Hepatic resistance	263	243	6	12	16.8 ± 3.7	17.0 ± 3.5	-0.48	0 (0)/4 (2)	0 (0)/5 (3)	0 (0)/5 (3)
Kronborg 2023^39^	Denmark	Prevention of decompensation/mortality	Atorvastatin *vs.* placebo	Hepatic resistance	78	78	6	6	15.1 ± 7.8	16.1	0.00	4 (10)/4 (11)	2 (5)/2 (5)	2 (5)/2 (5)
Ranjan 2024^40^	India	Primary prophylaxis	Propranolol + midodrine *vs.* propranolol	Hepatic inflow	140	53	3	12	18.0 ± 3.0	18.6 ± 3.7	-1.77	16 (23)/8 (11)	19 (27)/6 (9)	19 (27)/6 (9)
Tevethia 2024^41^	India	Primary prophylaxis	VBL + carvedilol *vs.* VBL	Hepatic inflow	220	159	12	12	17.5 ± 3.6	17.9 ± 3.6	-4.00	16 (15)/7 (6)	28 (25)/13 (12)	28 (25)/13 (12)
Khajuria 2025^42^	India	Prevention of further decompensation	Carvedilol *vs.* SMT (low salt diet ± anti-mineralocorticoid and furosemide)	Hepatic inflow	104	38	12	12	15.9 ± 2.2	14.9 ± 2.8	-6.90	13 (25)/5 (10)	28 (54)/11 (21)	9 (17)/4 (8)

ΔHVPG Diff = between-group difference in HVPG change (treatment minus control; negative values indicate greater reduction in the treatment group). Variceal events defined hierarchically as variceal progression (when reported) or variceal bleeding. BB, beta-blocker; C/T, control/treatment; Ctrl, control; FU, follow-up; HVPG, hepatic venous pressure gradient; mo, months; Mort, mortality;SMT, standard medical therapy; Trt, treatment; VBL, variceal band ligation.

**Fig. 1:**
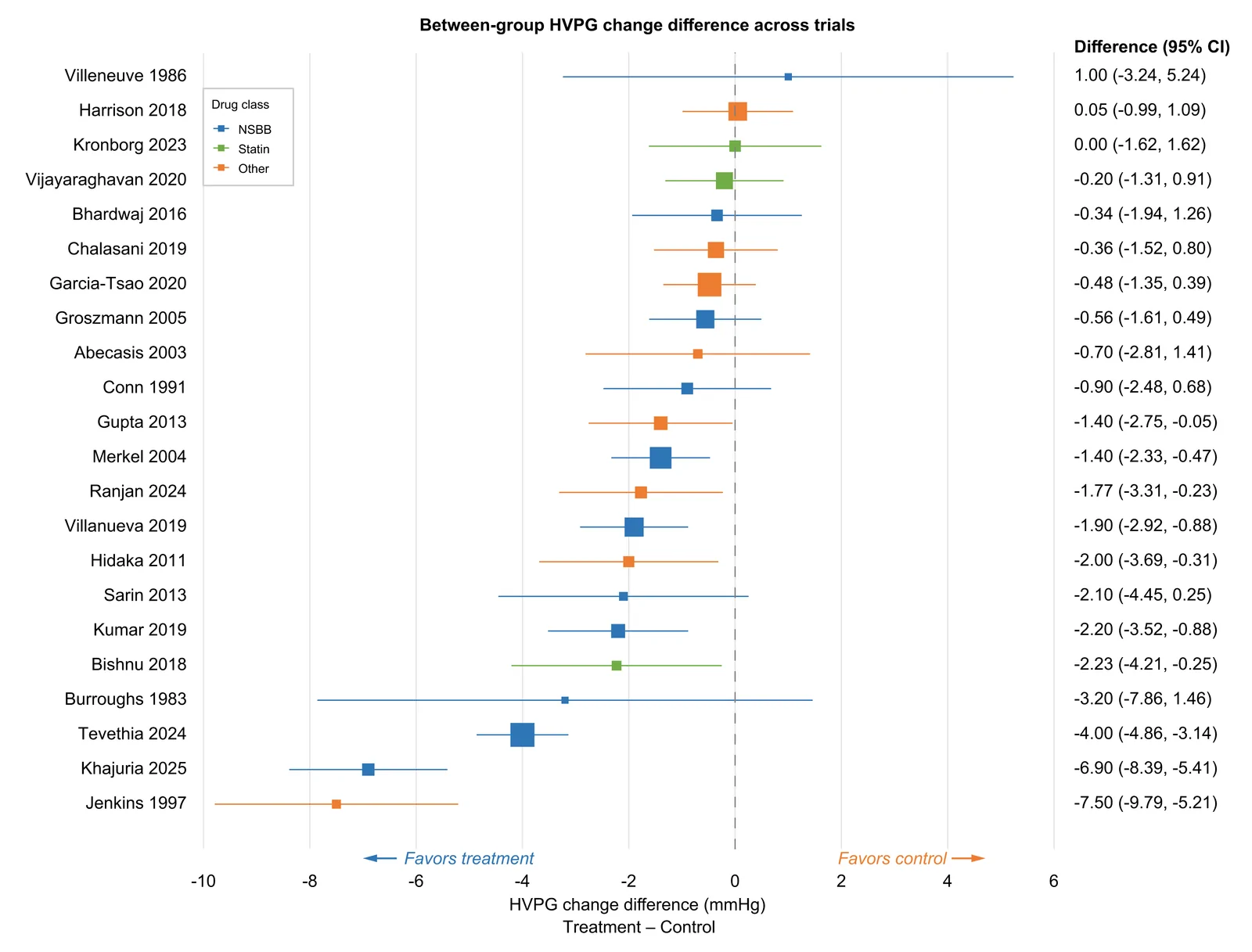
Forest plot of the between-group HVPG change difference (treatment minus control, in mmHg) across all 22 randomized controlled trials. Negative values indicate greater HVPG reduction with the experimental treatment. Square size is proportional to the inverse-variance weight of each study. Colors indicate drug class: blue = non-selective beta-blockers (NSBB), green = statins, orange = other agents. The dashed vertical line represents no difference (0 mmHg). The wide range of HVPG reductions (-7.5 to +1.0 mmHg) provides the spread in the predictor variable necessary for the meta-regression analysis. HVPG, hepatic venous pressure gradient.

### HVPG variance imputation

Of 22 studies, nine directly reported the SD of HVPG change from baseline. For the remaining 13, change SDs were calculated from baseline and follow-up SDs using the empirically derived correlation (ρ = 0.687; range across studies 0.50–0.95) in 12, and imputed from the median of reported studies in 1.^13^ This strategy recovered usable variance data for all 22 studies (Table S2).

### Primary outcomes

In random-effects meta-regression, each 1 mmHg greater HVPG reduction in the treatment *vs.* control group was associated with significantly lower all-cause mortality: OR 0.850 (95% CI 0.771–0.937; *p* = 0.004). There was no residual heterogeneity (*I*^2^ = 0%; τ^2^ = 0; Q = 9.2, *p* = 0.98). Across the 22 trials, 127 deaths occurred in treatment groups and 181 in control groups. The intercept was non-significant (log OR = -0.21, *p* = 0.11), indicating no detectable effect on mortality in the absence of a between-group HVPG difference. Fig. 2A shows the meta-regression plot for mortality.

**Fig. 2:**
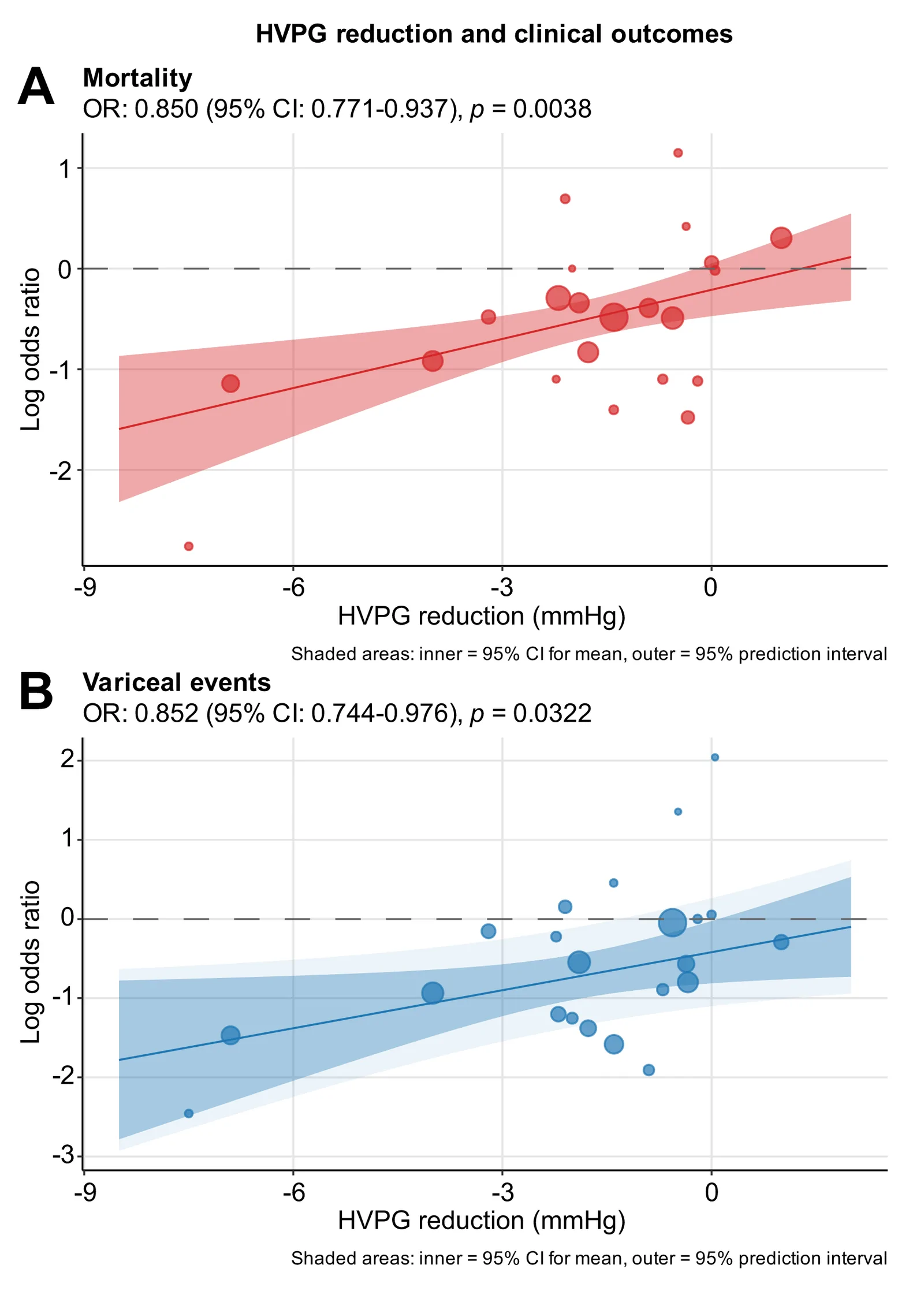
Meta-regression of HVPG reduction and clinical outcomes. (A) All-cause mortality. (B) Variceal events (hierarchical: variceal progression when reported, otherwise variceal bleeding). Each circle represents one RCT; circle size is proportional to the inverse variance of the log odds ratio (study precision). The x-axis shows the between-group difference in HVPG change (treatment minus control; more negative values indicate greater HVPG reduction in the treatment group). The y-axis shows the log odds ratio for the clinical outcome (negative values favor treatment). The solid line represents the fitted meta-regression line; inner shaded area represents the 95% confidence interval for the mean; outer shaded area represents the 95% prediction interval. In panel A both overlap. HVPG, hepatic venous pressure gradient; OR, odds ratio; RCT, randomized controlled trial.

Each 1 mmHg greater HVPG reduction was also associated with significantly fewer variceal events: OR 0.852 (95% CI 0.744–0.976; *p* = 0.032). Residual heterogeneity was low (*I*^2^ = 18.8%; τ^2^ = 0.071), and 44% of between-study variance was explained by HVPG reduction (R^2^ = 44.4%). There were 210 variceal events in treatment groups and 310 in control groups. The intercept was significant (log OR = -0.42, *p* = 0.038), suggesting that HVPG reduction as a surrogate might not fully capture the drug effect. Fig. 2B shows the meta-regression plot for variceal events.

### Exploratory analysis: variceal bleeding

In exploratory analysis, the association between HVPG reduction and variceal bleeding alone was directionally consistent but did not reach statistical significance: OR 0.871 per 1 mmHg (95% CI 0.741–1.024; *p* = 0.111; *I*^2^ = 7.3%) (Fig. 3). This wider confidence interval likely reflects both fewer events and the loss of information from trials reporting variceal progression but not bleeding as a separate endpoint.

**Fig. 3:**
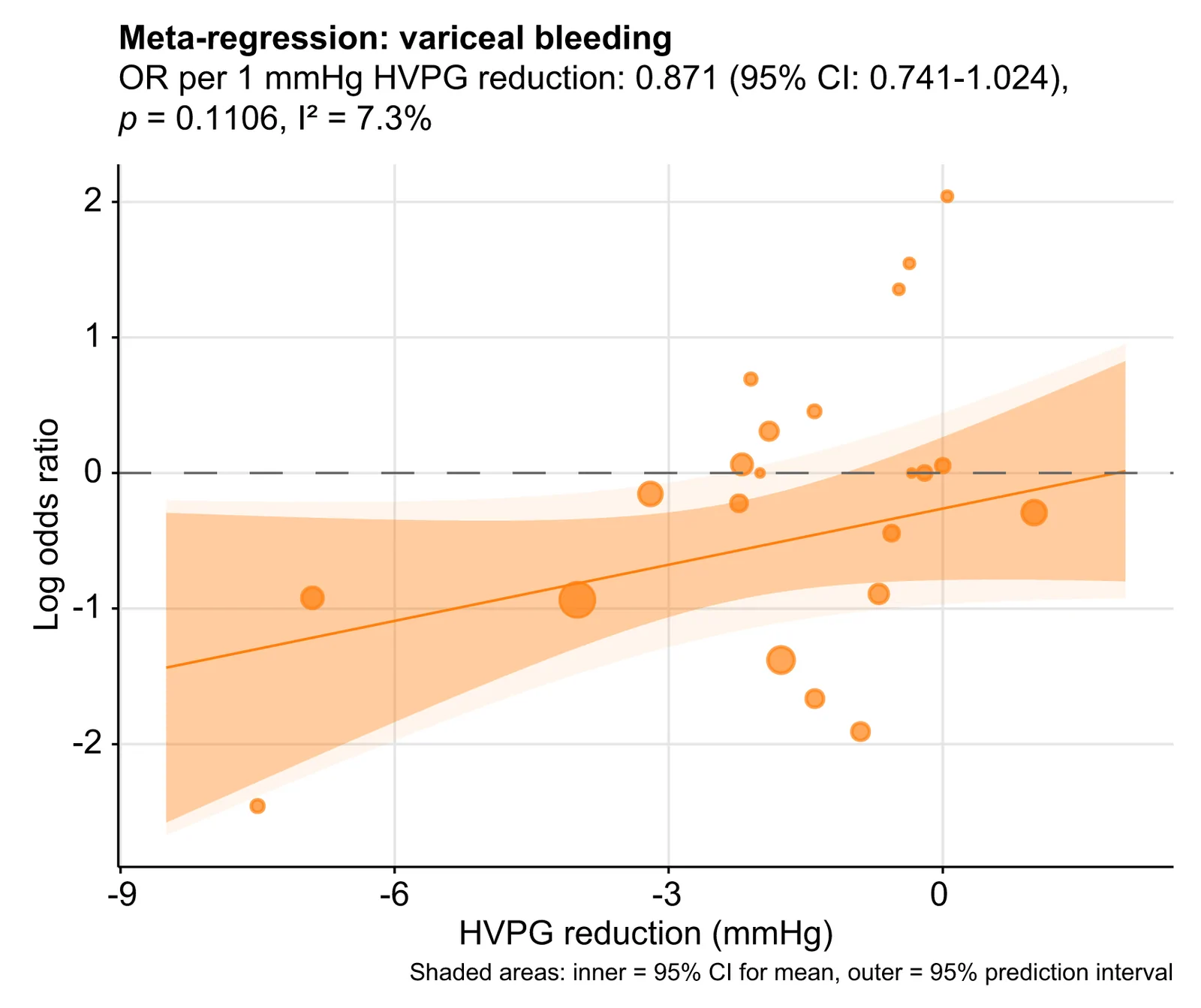
Meta-regression of HVPG reduction and variceal bleeding (exploratory analysis). Each circle represents one RCT; circle size is proportional to study precision. The association was directionally consistent with primary outcomes but did not reach statistical significance (OR 0.871 per 1 mmHg, 95% CI 0.741–1.024, *p* = 0.111). HVPG, hepatic venous pressure gradient; OR, odds ratio; RCT, randomized controlled trial.

### Potential for translation into trial design

Each 1 mmHg greater between-group HVPG reduction was associated with ∼15% lower odds of each outcome (all-cause mortality OR 0.850 per mmHg, 95% CI 0.771–0.937; variceal events OR 0.852, 95% CI 0.744–0.976). The effect implied by the surrogate slope alone, excluding the fitted intercept (hereafter the HVPG-attributable effect), therefore compounds multiplicatively: a treatment arm achieving a 2 mmHg greater reduction than control corresponds to an OR of 0.72 (95% CI 0.59–0.88) for mortality and 0.73 (95% CI 0.55–0.95) for variceal events, and at 3 mmHg to 0.61 (95% CI 0.46–0.82) and 0.62 (95% CI 0.41–0.93) respectively (Table S3A).

The fitted models additionally contain a trial-level intercept, that is, an average treatment effect at zero between-group HVPG difference. Adding this term gives the total effect predicted for a future trial. These predictions and their numeric 95% prediction intervals are shown in Table S3B. For all-cause mortality the estimated between-trial variance was zero (τ^2^ = 0), so the prediction interval coincides with the confidence interval (Fig. 2A). With 22 trials this estimate of τ^2^ is imprecise, and the mortality prediction intervals are therefore likely to understate the uncertainty a future trial would face. These intervals also describe the true effect expected in a new trial. The effect a new trial actually observes carries further sampling error that depends on its own sample size and event rate, and this must be added separately when planning. Because a trial-level design cannot identify what the intercept represents, we regard the slope-based estimates (Table S3A) as the figures a development program should plan against, and the intercept-inclusive predictions as the larger effect that would be expected only if that intercept also transfers to a new trial. The HVPG reductions considered here all lie within the range observed across the included trials, so none of these estimates requires extrapolation.

### Sensitivity analyses

Results were robust across all alternative weighting schemes. For mortality, the OR per 1 mmHg ranged from 0.850 (standard outcome-based weighting) to 0.810 (predictor-based weighting), with joint weighting (OR 0.857) and iteratively reweighted (OR 0.856) estimates closely matching the primary analysis. For variceal events, the pattern was similar: outcome-based OR 0.852, predictor-based OR 0.734, joint OR 0.849, iterative OR 0.849 (Table S4).

Because ρ enters only the predictor variance, the outcome-weighted primary analysis is invariant to ρ by construction; ρ affects only the predictor-weighted and joint schemes, which varied minimally across ρ = 0.5–0.8 (Table S5). Leave-one-out analysis demonstrated stability, and no single study altered statistical significance for mortality. For variceal events, exclusion of three individual studies^29^^,^^32^^,^^42^ shifted *p* values above 0.05, although effect estimates remained directionally consistent (Table S6). In exploratory analyses, the non-linear quadratic model did not fit better than the linear model, indicating no evidence against linearity within the observed range. A one-stage binomial-normal model handling zero-event arms without continuity correction gave materially unchanged estimates (mortality OR 0.856; variceal events OR 0.853) (Table S7). Influence diagnostics (Cook’s distance) identified no dominant study (all Cook’s D <1 for every outcome; Table S8). Correcting the slope for measurement error in the predictor (SIMEX) moved both estimates slightly further from the null (mortality OR 0.823, 95% CI 0.676–0.921, variceal events OR 0.832, 95% CI 0.665–0.955), corresponding to attenuation of the naive slope of 20% and 15% respectively; scenarios based on our previously published external test–retest variance of HVPG change gave similar results (Table S9).

## Discussion

In this meta-regression of 22 randomized controlled trials including 2,871 patients with portal hypertension, we demonstrate a trial-level surrogate relationship between the magnitude of HVPG reduction and clinical outcomes. Each 1 mmHg greater placebo-corrected reduction in HVPG was associated with approximately 15% lower odds of both all-cause mortality and variceal events, with low residual heterogeneity and concordant results across multiple analytical approaches. These findings provide a quantitative estimate of the clinical return per unit reduction in portal pressure at the trial level.

The consistency of this relationship across outcomes and sensitivity analyses further supports the validity of the observed surrogacy. The association between HVPG reduction and mortality showed minimal residual heterogeneity, suggesting a broadly consistent relationship across trials despite differences in patient populations, pharmacological interventions, and follow-up durations. However, with only 22 studies, the power to detect between-study variability remains limited, and therefore the low *I*^2^ should not be interpreted as evidence of true homogeneity. The association with variceal events further supports HVPG reduction as a trial-level surrogate for treatment benefit, while the weaker association observed for variceal bleeding alone likely reflects reduced statistical power and the influence of post-randomization interventions, such as endoscopic therapy following variceal progression.^17^

The use of RCTs rather than observational data addresses the confounding inherent in prior studies:^6^ randomization creates groups comparable in prognosis, and by using between-group differences as the unit of analysis and we neutralize the influence of spontaneous HVPG fluctuations.^10^ The meta-regression framework applied here parallels approaches used in other fields to evaluate biomarker-outcome relationships at the trial level^7^ It is important to note that conventional meta-regression assumes that the predictor is measured without error. In the present context, HVPG change is estimated in subsets of randomized patients and is subject to measurement variability, introducing potential attenuation in the associations (which the SIMEX correction indicates is modest) so the primary estimates are likely to be conservative.

These findings support HVPG reduction as a candidate trial-level surrogate: across trials, the treatment effect on HVPG accounted for a substantial part of the between-trial variation in the treatment effect on clinical outcomes (R^2^ = 44% for variceal events), but not all of it.^7^^,^^43^ The intercept in the variceal events model was not zero, that is, a treatment effect was apparent even when the placebo-corrected HVPG change was zero, indicating that the trial-level slope alone does not account for the whole of the observed effect. The simplest explanation for this finding is measurement imprecision in HVPG assessment^10^ that attenuates the observed slope and shifts effect to the intercept, although pressure-independent drug effects or beneficial effects on hepatic blood flow not captured by HVPG decrease cannot be excluded.^44^^,^^45^ For mortality, the intercept was not statistically distinguishable from zero; we interpret both intercepts descriptively only and do not treat them as evidence of a pressure-independent causal effect, which this trial-level design cannot identify.

These results have practical implications for clinical trial design. A drug that achieves a 2-mmHg placebo-corrected HVPG reduction in phase II would be predicted to have a treatment-effect OR of approximately 0.72 for mortality and 0.73 for variceal events, effect sizes that can inform sample size calculations and go/no-go decisions for phase III programs, on the assumption that a new drug effect on outcomes relates to its effect on HVPG as in the trials analyzed here. Conversely, drugs with minimal HVPG effects would be predicted to have limited clinical benefit through hemodynamic mechanisms (which does not negate other potential effects, for example, on liver function). The quantitative framework we provide could thus improve the efficiency of drug development in portal hypertension, a field where phase III trials require large sample sizes and long follow-up because of relatively low event rates.

A practical question for translating these findings to phase III trial design is the relationship between the ORs reported here and the HRs typically used as the primary efficacy measure in time-to-event analyses. For rare events, where event rates are below 15%, the OR closely approximates both the risk ratio and the HR. For more common events, the OR overestimates the relative risk. Combining these trial-level estimates with established HVPG test–retest reliability data can further inform power calculations for phase III trials that use clinical endpoints.^10^

It is important to note that HVPG was typically measured in a subset of randomized patients (Table 1). If patients experiencing early clinical events were systematically excluded from hemodynamic assessment, this could introduce bias. However, such bias would likely tend to attenuate the observed association by reducing variance in the HVPG predictor, suggesting our estimates may be conservative. Earlier HVPG measurement time points may mitigate this concern by capturing hemodynamic responses before many clinical events occur.

Several limitations of this analysis deserve discussion. The most relevant for interpretation is that the ecological design precludes patient-level inference; the relationship between HVPG reduction and outcomes at the trial level need not hold at the individual level. Second, variance imputation was necessary for most studies, although results were insensitive to the correlation assumption and to exclusion of imputed studies. Third, heterogeneity in HVPG measurement protocols, timing, and the proportion of randomized patients undergoing hemodynamic assessment introduces variability that may attenuate the observed relationship. However, our results were consistent across sensitivity analyses explicitly accounting for predictor measurement error, suggesting that attenuation bias from measurement noise is modest. Fourth, other outcomes such as ascites were reported by too few trials to support a reliable analysis. Fifth, with only 22 trials, power for subgroup analyses analyzing different drug classes (beta-blockers accounted for most studies), different clinical contexts (compensated *vs*. decompensated patients) and detection of non-linear relationships is limited.

In conclusion, we demonstrate a trial-level surrogate relationship between HVPG reduction and clinical outcomes across RCTs assessing pharmacological treatments for portal hypertension, with approximately 15% lower odds of mortality and variceal events per 1 mmHg of portal pressure reduction. These findings support HVPG reduction as a trial-level surrogate that predicts treatment benefit and provide a quantitative basis for translating phase II hemodynamic responses into expected phase III clinical outcomes.

## Abbreviations

BB, beta-blocker; C/T, control/treatment; Ctrl, control; FU, follow-up; HVPG, hepatic venous pressure gradient; mo, months; Mort, mortality; OR, odds ratio; PRISMA, Preferred Reporting Items for Systematic Review and Meta-Analysis; RCT, randomized controlled trial; SMT, standard medical therapy; Trt, treatment; VBL, variceal band ligation.

## Authors’ contributions

Conceptualization: MT, IP, DVvZ, YJW, JGA. Methodology (search strategy design and execution): JYK, YJW, JGA. Resources: JYK. Investigation: MT, IP, JJRT, DVvZ, BK, SS. Data curation: MT, IP, JJRT, DVvZ, BK, SS. Formal analysis: MT, IP, YJW, JGA. Supervision: YJW, JGA. Writing—original draft: MT, IP. Writing—review and editing: JJRT, DVvZ, BK, SS, JYK, YJW, JGA.

## Data availability

The trial-level dataset extracted for this review is available in the tables, and the R code used for all analyses are available from the corresponding author on reasonable request.

## Declaration of generative AI and AI-assisted technologies in the writing process

During the preparation of this work, the authors used Claude (Anthropic) to assist with refreshing numerical values in the manuscript and supplementary tables after data corrections and re-analysis, with reviewing the R analysis code, and with cross-checking selected meta-regression sub-statistics against the *metafor* output. The authors reviewed and edited the output as needed and take full responsibility for the content of the published article.

## Financial support

This research did not receive any specific grant from funding agencies in the public, commercial, or not-for-profit sectors.

## Conflicts of interest

JGA reports consulting fees from Madrigal, Boehringer Ingelheim, 89Bio, Novo Nordisk, AstraZeneca, Boston Pharmaceuticals, Agomab, Biossil, StatelyBio, Bausch Health, Roche, HepQuant, and AbbVie; stock options from Biossil; speaker fees from Terumo, Ferring, and Echosens; grant support from Gilead, paid to the institution; and clinical trial support from Surrozen, Boehringer Ingelheim, and Salix, paid to the institution. YJW reports grant support from Gilead Sciences, Roche, Changi General Hospital, and the Medicine Academic Clinical Program, SingHealth; lecture fees from Gilead Sciences, Ipsen, and Ferring; and advisory board fees from Boehringer Ingelheim and Ipsen. Other authors: no conflicts.

Please refer to the accompanying ICMJE disclosure forms for further details.
