# Supplementary material for: Magnitude of portal pressure reduction and clinical outcomes in randomized trials
Source: JHEP Rep. 2026 Sep 21;8(12):102028. doi: 10.1016/j.jhepr.2026.102028 (PMC13756290; doi:10.1016/j.jhepr.2026.102028)
Supplement: Supplementary file 1 [file mmc1.pdf]

Magnitude of portal pressure reduction and clinical outcomes in randomized trials

Matthew Tam, Irene Pascual, Jarell Jie-Rae Tan, Daniel Veldhuijzen van Zanten, Beverley Kok, Shuen Sung, Janice Y. Kung, Yu Jun Wong, Juan G. Abraldes

Table of contents

Supplementary Methods: Search Strategy 2

Appendix 1A: MEDLINE Search Strategy 3

Appendix 1B: EMBASE Search Strategy 4

Appendix 1C: Cochrane Library Search Strategy 6

Appendix 1D: Scopus Search Strategy 7

Fig. S1 8

Fig. S2 9

Table S1 10

Table S2 11

Table S3 12

Table S4 14

Table S5 15

Table S6 17

Table S7 18

Table S8 19

Table S9 23

Supplementary references 25

Supplementary Methods: Search Strategy

The medical librarian (JK) designed comprehensive searches in MEDLINE (via Ovid), EMBASE (via Ovid), Scopus, and Cochrane Library (via Wiley). In order to identify all potential drug interventions to prevent clinical complications in cirrhosis, a comprehensive list of drug names were used in the search strategy including all relevant keywords and controlled vocabulary. All searches were limited to English language. In addition to subscription databases, the research team searched for grey literature by searching key websites that may have publications on the topic. Bibliographies from included studies were also reviewed. The original search was conducted on July 19, 2020 and was subsequently updated using the same search strategies, with the final update conducted in December 2025. Title and abstract screening and full text screening were conducted by two independent reviewers.

Appendix 1A: MEDLINE Search Strategy

Database: Ovid MEDLINE(R) ALL 1946 to July 17, 2020 (updated December 2025)

#	Search Term
1	cirrho*.mp.
2	(acute on chronic liver failure or acute on chronic hepatic failure).mp.
3	or/1-2
4	exp Adrenergic beta-Antagonists/
5	(beta blocker* or beta adren* or beta antagonists).mp.
6	(atenolol or tenormin).mp.
7	(carvedilol or coreg).mp.
8	(labetalol or normodyne or trandate).mp.
9	(metoprolol or lopressor or metolar or toprol-XL).mp.
10	(nadolol or corgard).mp.
11	(nebivolol or bystolic).mp.
12	(penbutolol or levatol).mp.
13	(propranolol or inderal or innopran).mp.
14	(sotalol or betaspace or sorine or sotylize).mp.
15	(timol* or blocadren or apo-timol).mp.
16	exp Hydroxymethylglutaryl-CoA Reductase Inhibitors/
17	statins.mp.
18	(atorvastatin or vastatin or lipitor).mp.
19	(fluvastatin or lescol).mp.
20	(lovastatin or altoprev or mevacor).mp.
21	(pitavastatin or livalo).mp.
22	(pravastatin or pravachol).mp.

23	(rosuvastatin or crestor or ezallor).mp.
24	(simvastatin or flolipid or zocor).mp.
25	(prazosin or minipress).mp.
26	(nitrates or nitric oxide).mp.
27	((drug* or pharmaceut* or pharmacolog*) adj1 (intervention* or treatment* or therap* or program*)).ti,ab.
28	*Drug Therapy/ or exp *Drug Prescriptions/ or (pharmacotherap* or pharmacotreatment).ti,ab.
29	or/4-28
30	exp Portal Pressure/
31	exp Hypertension, Portal/
32	((portal adj2 pressure) or portal hypertension*).mp.
33	(hepatic venous pressure gradient or HVPG or wedged hepatic venous pressure or WHVP or hepatic vein* or liver vein*).mp.
34	or/30-33
35	3 and 29 and 34
36	randomized controlled trial.pt.
37	clinical trial.pt.
38	randomi?ed.ti,ab.
39	placebo.ti,ab.
40	dt.fs.
41	randomly.ti,ab.
42	trial.ti,ab.
43	groups.ti,ab.
44	or/36-43
45	animals/
46	humans/
47	45 not (45 and 46)
48	44 not 47 [RCT filter]
49	35 and 48
50	limit 49 to english language
51	limit 50 to meta analysis
52	limit 50 to "systematic review"
53	limit 50 to "review"
54	limit 50 to comment
55	limit 50 to editorial
56	or/51-55
57	50 not 56

Appendix 1B: EMBASE Search Strategy

Database: Ovid Embase 1974 to July 16, 2020 (updated December 2025)

#	Search Term
1	cirrho*.mp.
2	(acute on chronic liver failure or acute on chronic hepatic failure).mp.
3	or/1-2
4	exp beta adrenergic receptor blocking agent/
5	(beta blocker* or beta adren* or beta antagonists).mp.
6	(atenolol or tenormin).mp.
7	(carvedilol or coreg).mp.
8	(labetalol or normodyne or trandate).mp.
9	(metoprolol or lopressor or metolar or toprol-XL).mp.
10	(nadolol or corgard).mp.
11	(nebivolol or bystolic).mp.
12	(penbutolol or levatol).mp.
13	(propranolol or inderal or innopran).mp.
14	(sotalol or betaspace or sorine or sotylize).mp.
15	(timol* or blocadren or apo-timol).mp.
16	exp hydroxymethylglutaryl coenzyme A reductase inhibitor/
17	statins.mp.
18	(atorvastatin or vastatin or lipitor).mp.
19	(fluvastatin or lescol).mp.
20	(lovastatin or altoprev or mevacor).mp.
21	(pitavastatin or livalo).mp.
22	(pravastatin or pravachol).mp.
23	(rosuvastatin or crestor or ezallor).mp.
24	(simvastatin or flolipid or zocor).mp.
25	(prazosin or minipress).mp.
26	(nitrates or nitric oxide).mp.
27	((drug* or pharmaceut* or pharmacolog*) adj1 (intervention* or treatment* or therap* or program*)).ti,ab.
28	*drug therapy/ or exp *prescription/ or (pharmacotherap* or pharmacotreatment).ti,ab.
29	or/4-28
30	exp portal vein blood pressure/
31	exp portal hypertension/
32	((portal adj2 pressure) or portal hypertension*).mp.
33	(hepatic venous pressure gradient or HVPG or wedged hepatic venous pressure or WHVP or hepatic vein* or liver vein*).mp.

34	or/30-33
35	3 and 29 and 34
36	exp clinical trial/
37	randomi?ed.ti,ab.
38	placebo.ti,ab.
39	dt.fs.
40	randomly.ti,ab.
41	trial.ti,ab.
42	groups.ti,ab.
43	or/36-42
44	animal/
45	human/
46	44 not (44 and 45)
47	43 not 46
48	35 and 47
49	limit 48 to english language
50	limit 49 to meta analysis
51	limit 49 to "systematic review"
52	limit 49 to "review"
53	limit 49 to editorial
54	or/50-53
55	49 not 54

Appendix 1C: Cochrane Library Search Strategy

Database: Cochrane Library (via Wiley), Trials

#1 cirrho* or [mh cirrhosis]

#2 "acute on chronic liver failure" or "acute on chronic hepatic failure"

#3 MeSH descriptor: [Acute-On-Chronic Liver Failure] explode all trees

#4 #1 or #2 or #3

#5 [mh "Adrenergic beta-Antagonists"]

#6 beta NEXT blocker* or beta NEXT adren* or "beta antagonists"

#7 atenolol or tenormin

#8 carvedilol or coreg

#9 labetalol or normodyne or trandate

#10 metoprolol or lopressor or metolar or toprol-XL

#11 nadolol or corgard

#12 nebivolol or bystolic

#13 penbutolol or levatol

#14 propranolol or inderal or innopran

#15 sotalol or betaspace or sorine or sotylize

#16 timol* or blocadren or apo-timol

#17 [mh "Hydroxymethylglutaryl-CoA Reductase Inhibitors"]

#18 statins

#19 atorvastatin or vastatin or lipitor

#20 fluvastatin or lescol

#21 lovastatin or altoprev or mevacor

#22 pitavastatin or livalo

#23 pravastatin or pravachol

#24 rosuvastatin or crestor or ezallor

#25 simvastatin or flolipid or zocor

#26 prazosin or minipress

#27 nitrates or "nitric oxide"

#28 (drug* or pharmaceut* or pharmacolog*) NEAR/1 (intervention* or treatment* or therap* or program*)

#29 [mh "drug therapy"] or [mh "drug prescriptions"]

#30 (pharmacotherap* or pharmacotreatment):ti,ab

#31 {or #5-#30}

#32 [mh "portal pressure"]

#33 [mh "hypertension, portal"/dt]
#34 ((portal NEAR/2 pressure) or portal NEXT hypertension*):ti,ab
#35 "hepatic venous pressure gradient" or HVPG or "wedged hepatic venous pressure" or WHVP or hepatic NEXT vein* or liver NEXT vein*
#36 {or #32-#35}
#37 #4 and #31 and #36

Appendix 1D: Scopus Search Strategy

Database: Scopus

(TITLE-ABS-KEY(cirrho* OR "acute on chronic liver failure" OR "acute on chronic hepatic failure") AND TITLE-ABS-KEY("beta blocker*" OR "beta adren*" OR "beta antagonists" OR atenolol OR tenormin OR carvedilol OR coreg OR labetalol OR normodyne OR trandate OR metoprolol OR lopressor OR metolar OR toprol-XL OR nadolol OR corgard OR nebivolol OR bystolic OR penbutolol OR levatol OR propranolol OR inderal OR innopran OR sotalol OR betaspace OR sorine OR sotylize OR timol* OR blocadren OR apo-timol OR "Hydroxymethylglutaryl-CoA Reductase Inhibitor*" OR statins OR atorvastatin OR vastatin OR lipitor OR fluvastatin OR lescol OR lovastatin OR altoprev OR mevacor OR pitavastatin OR livalo OR pravastatin OR pravachol OR rosuvastatin OR crestor OR ezallor OR simvastatin OR flolipid OR zocor OR prazosin OR minipress OR nitrates OR "nitric oxide" OR ((drug* OR pharmaceut* OR pharmacolog*) W/1 (intervention* OR treatment* OR therap* OR program*)) OR pharmacotherap* OR pharmacotreatment) AND TITLE-ABS-KEY((portal W/1 pressure) OR "portal hypertension*" OR "hepatic venous pressure gradient" OR HVPG OR "wedged hepatic venous pressure" OR WHVP OR "hepatic vein*" OR "liver vein*")) AND NOT TITLE(veterinary OR rabbit OR rabbits OR animal OR animals OR mouse OR mice OR rodent OR rodents OR rat OR rats OR hamster* OR pig OR pigs OR porcine OR horse* OR equine OR cow OR cows OR bovine OR goat OR goats OR sheep OR ovine OR canine OR dog OR dogs OR feline OR cat OR cats OR zebrafish) AND (EXCLUDE (DOCTYPE,"re") OR EXCLUDE (DOCTYPE,"le") OR EXCLUDE (DOCTYPE,"cp") OR EXCLUDE (DOCTYPE,"ch") OR EXCLUDE (DOCTYPE,"ed") OR EXCLUDE (DOCTYPE,"no") OR EXCLUDE (DOCTYPE,"sh") OR EXCLUDE (DOCTYPE,"er")) AND (LIMIT-TO (LANGUAGE,"English"))

Fig. S1: PRISMA Flow Diagram

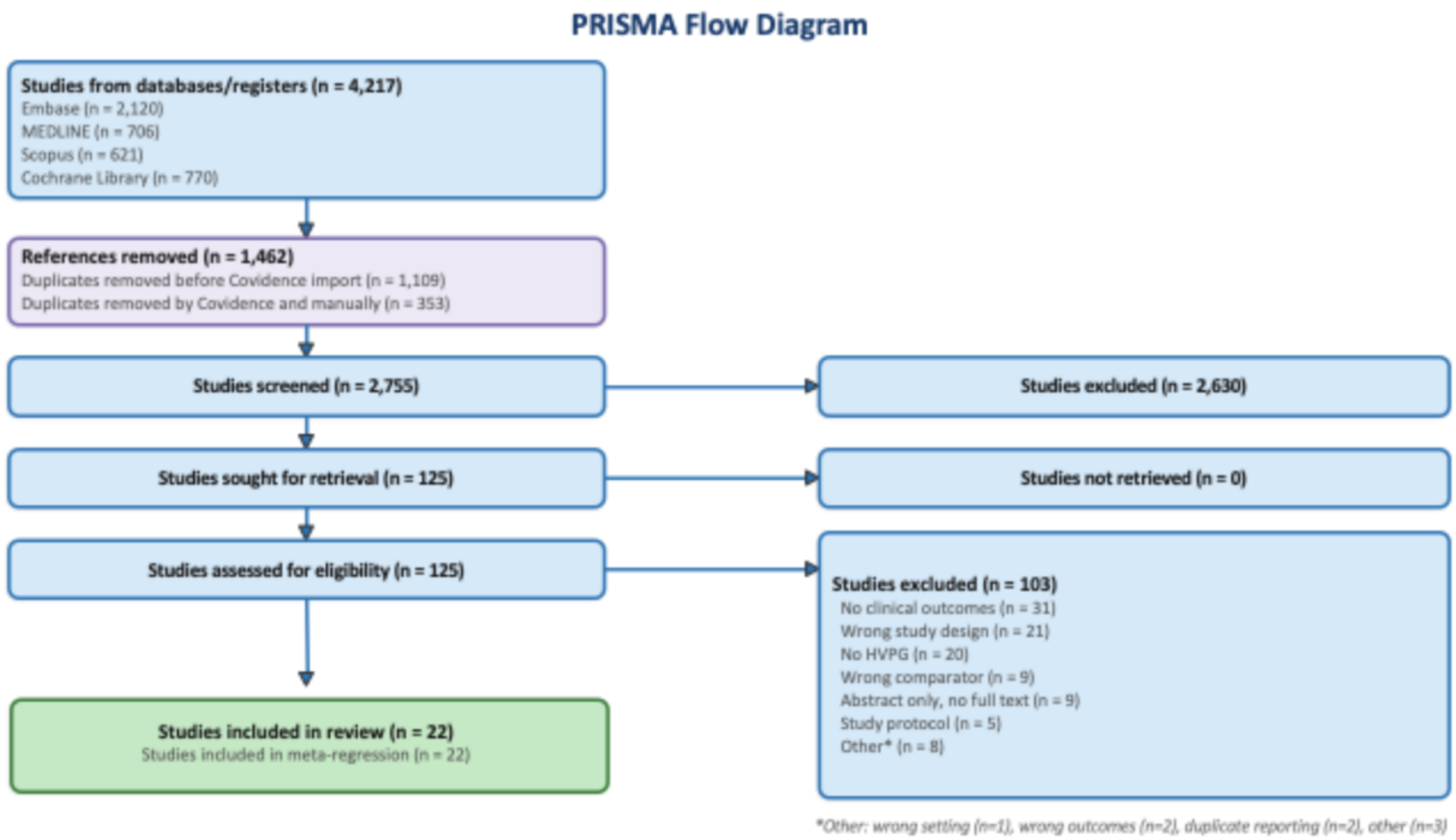

Fig. S2: Non-Linear Meta-Regression Analysis

A. Mortality

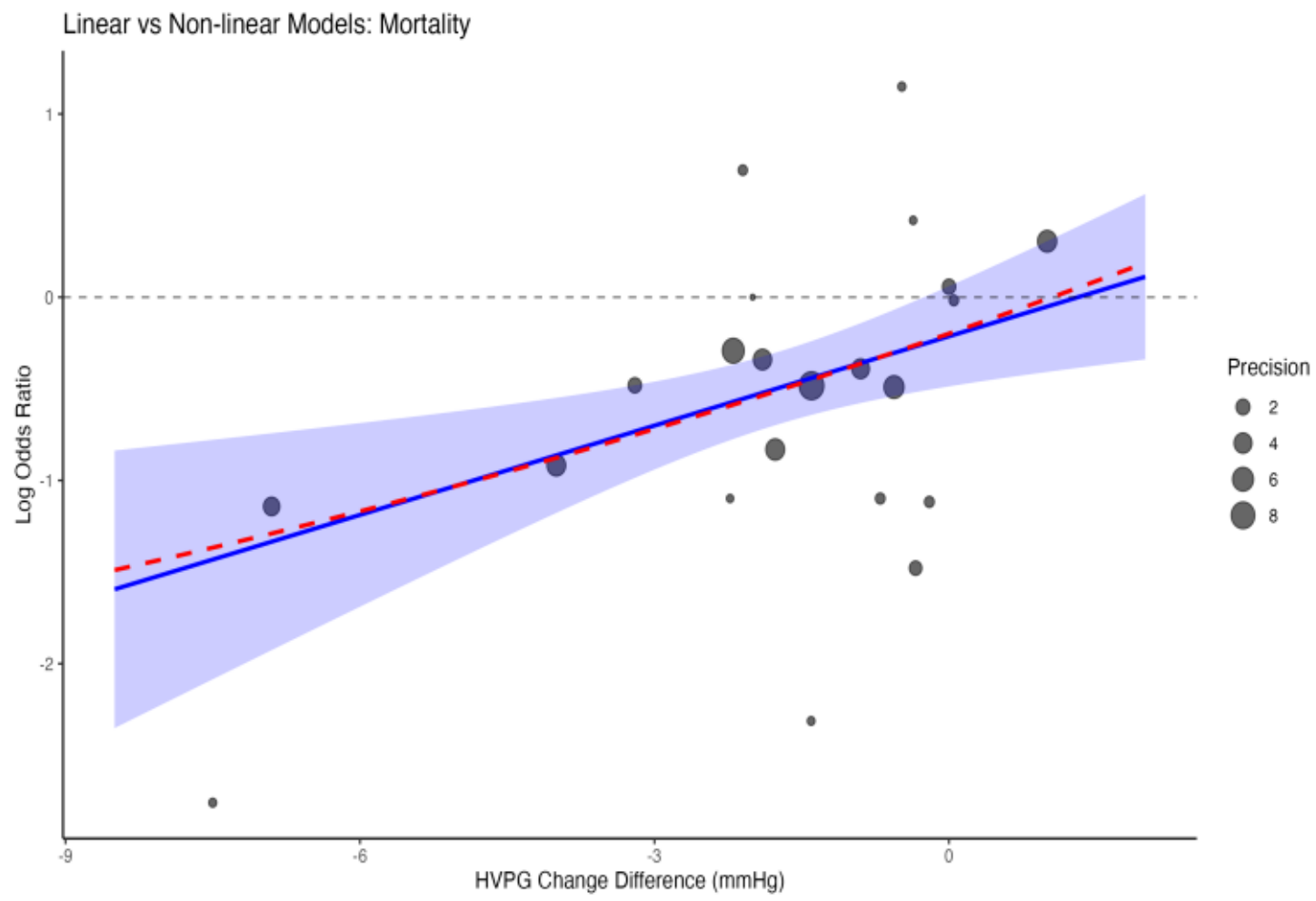

B. Variceal Events

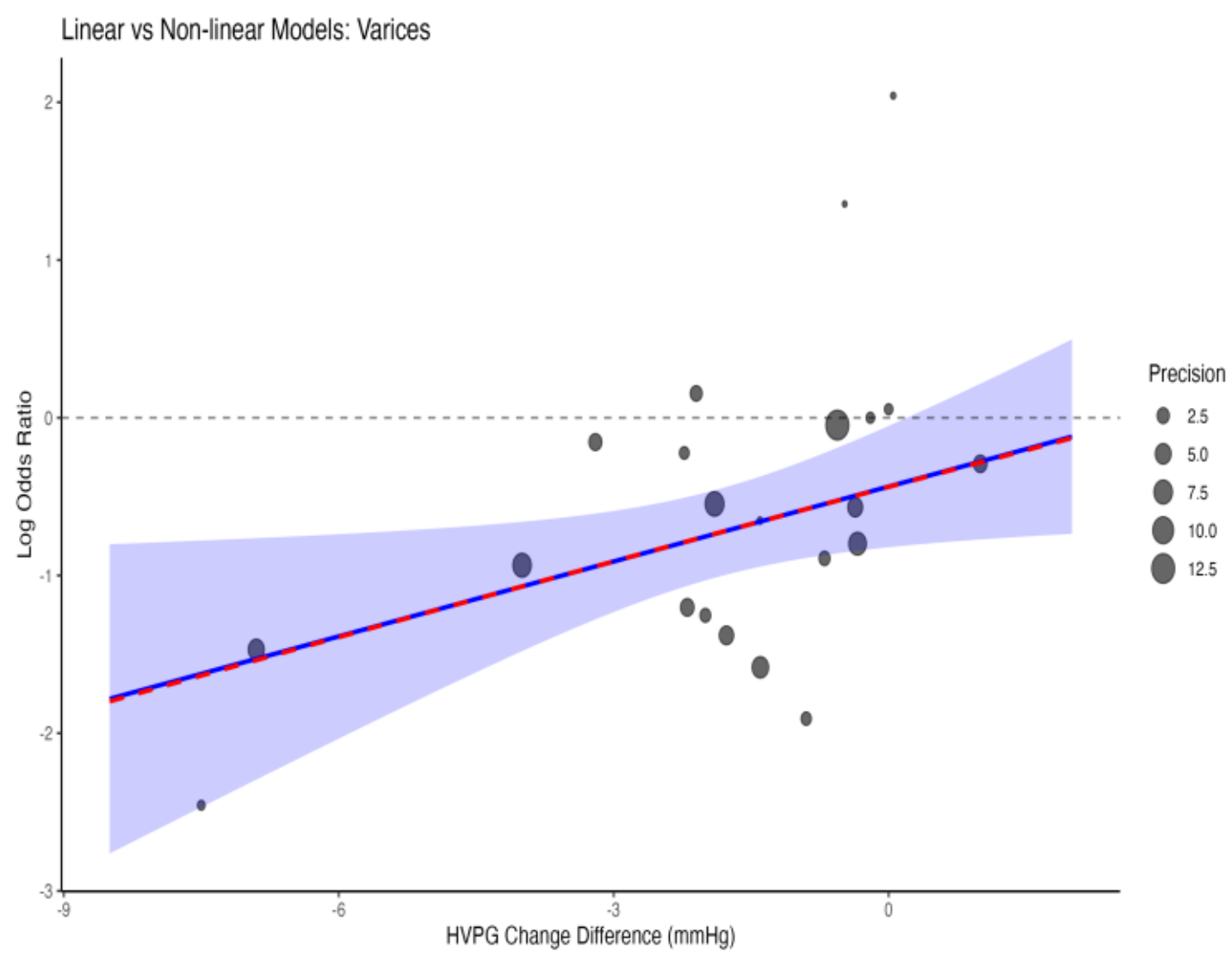

Fig. S2. *Comparison of linear and non-linear meta-regression models. (A) Mortality. (B) Variceal events. Blue solid line: linear model; red dashed line: quadratic model; blue shaded area: 95% confidence band for the linear model. Non-linear models did not improve fit (ΔAIC < 2 for all comparisons), consistent with a linear model within the observed range; ΔAIC < 2 indicates the models are statistically indistinguishable, not that linearity is confirmed.*

Table S1: Risk of Bias Assessment (RoB 2)

Risk of bias was assessed using the Cochrane Risk of Bias 2 (RoB 2) tool. Colors indicate: green = Low risk, yellow = Some concerns, red = High risk.

Study	Randomization	Deviations	Missing Data	Measurement	Reporting	Overall
Burroughs 1983	Some concerns	Some concerns	Low	Some concerns	Some concerns	Some concerns
Villeneuve 1986	Some concerns	Some concerns	High	Some concerns	Low	High
Conn 1991	Low	Low	Low	Low	Low	Low
Jenkins 1997	Low	High	Low	Some concerns	Low	High
Abecasis 2003	Low	Low	Low	Low	Low	Low
Merkel 2004	Low	Some concerns	Some concerns	Low	Low	Some concerns
Groszmann 2005	Low	Low	Some concerns	Low	Low	Some concerns
Hidaka 2011	Some concerns	High	Low	Low	Some concerns	High
Gupta 2013	Low	Low	Some concerns	Low	Low	Some concerns
Sarin 2013	Low	Some concerns	Low	Low	Low	Some concerns
Bhardwaj 2016	Low	Some concerns	Low	Low	Low	Some concerns
Bishnu 2018	Some concerns	Some concerns	Some concerns	Some concerns	Some concerns	High
Harrison 2018	Low	Low	Low	Low	Low	Low
Kumar 2019	Low	Low	Low	Low	Low	Low
Villanueva 2019	Low	Low	Low	Low	Low	Low
Chalasani 2019	Some concerns	Low	Low	Low	Low	Some concerns
Vijayaraghaven 2020	Low	Some concerns	High	Low	Low	High
Garcia-Tsao 2020	Low	Low	Low	Low	Low	Low
Kronborg 2023	Low	Low	Some concerns	Low	Low	Some concerns
Ranjan 2024	Low	Some concerns	Low	Some concerns	Low	Some concerns
Tevethia 2024	Low	Some concerns	Low	Some concerns	Low	Some concerns
Khajuria 2025	Low	Some concerns	Some concerns	Low	Low	Some concerns

Risk of bias assessed using the RoB 2 tool [1].

Table S2: HVPG Variance Imputation Details

Details of standard deviation sources for HVPG change from baseline. When SDs were not directly reported, they were calculated from baseline and follow-up SDs using the formula $SD\_change = \sqrt{(SD\_baseline^2 + SD\_followup^2 - 2\rho \times SD\_baseline \times SD\_followup)}$, with empirical $\rho = 0.687$. For Kumar 2019, SDs were imputed using the median from other studies.

Study	N (Ctrl)	N (Trt)	SD Ctrl	Source	SD Trt	Source	SE Diff	ΔHVPG
Ranjan 2024	30	23	2.87	Reported	2.81	Reported	0.786	-1.77
Kronborg 2023	40	38	3.80	Reported	3.50	Reported	0.827	0.00
Gupta 2013	26	49	2.70	Reported	3.09	Reported	0.690	-1.40
Vijayaraghaven 2020	62	59	3.34	Calculated	2.90	Calculated	0.567	-0.20
Garcia-Tsao 2020	64	179	3.00	Reported	3.50	Reported	0.457	-0.48
Kumar 2019	46	45	2.87	Imputed	3.50	Imputed	0.672	-2.20
Burroughs 1983	9	10	6.23	Calculated	3.65	Calculated	2.377	-3.20
Abecasis 2003	12	12	2.85	Calculated	2.40	Calculated	1.077	-0.70
Villeneuve 1986	8	11	4.58	Calculated	4.74	Calculated	2.162	1.00
Merkel 2004	9	10	0.97	Calculated	1.09	Calculated	0.473	-1.40
Sarin 2013	24	25	4.18	Calculated	4.23	Calculated	1.201	-2.10
Bishnu 2018	12	11	1.88	Reported	2.82	Reported	1.009	-2.23
Conn 1991	39	45	4.12	Calculated	3.10	Calculated	0.805	-0.90
Groszmann 2005	82	72	3.14	Calculated	3.49	Calculated	0.538	-0.56
Bhardwaj 2016	48	52	3.91	Calculated	4.23	Calculated	0.814	-0.34
Villanueva 2019	78	78	3.23	Calculated	3.23	Calculated	0.518	-1.90
Jenkins 1997	16	16	2.59	Reported	3.89	Reported	1.168	-7.50
Hidaka 2011	23	22	2.78	Calculated	2.98	Calculated	0.860	-2.00
Chalasani 2019	51	100	3.53	Reported	3.63	Reported	0.613	-0.36
Harrison 2018	85	173	4.01	Reported	3.99	Reported	0.530	0.05
Tevethia 2024	79	80	2.89	Calculated	2.65	Calculated	0.440	-4.00
Khajuria 2025	18	20	2.20	Reported	2.48	Reported	0.759	-6.90

N = number of patients with paired HVPG measurements; SD = standard deviation of HVPG change (mmHg); SE Diff = standard error of the between-group HVPG change difference; ΔHVPG = treatment minus control HVPG change difference (mmHg, negative = greater reduction with treatment).

Table S3. Trial-level treatment effect at selected HVPG reductions

A. Effect attributable to the HVPG reduction (surrogate slope only)

Outcome	Between-group HVPG reduction (mmHg)	OR	95% CI
All-cause mortality	1	0.850	0.771–0.937
	2	0.722	0.594–0.878
	3	0.614	0.458–0.822
Variceal events	1	0.852	0.744–0.976
	2	0.726	0.553–0.954
	3	0.619	0.411–0.931

Odds ratio obtained by compounding the per-mmHg surrogate slope. The confidence limits are the per-mmHg confidence limits raised to the same power. This is the component of the trial-level treatment effect attributable to the HVPG reduction itself, and excludes the model intercept.

B. Total effect predicted for a future trial (slope plus intercept)

Outcome	Between-group HVPG reduction (mmHg)	Predicted OR	95% CI	95% prediction interval
All-cause mortality	1	0.688	0.561–0.844	0.561–0.844
	2	0.585	0.484–0.708	0.484–0.708
	3	0.497	0.395–0.625	0.395–0.625
Variceal events	1	0.560	0.411–0.764	0.296–1.060
	2	0.477	0.359–0.634	0.255–0.892
	3	0.407	0.293–0.564	0.213–0.776

Predicted odds ratio for a future trial achieving the stated between-group HVPG reduction, read from the fitted meta-regression line and therefore including the model intercept as well as the slope. For all-cause mortality the estimated between-trial variance was zero ($\tau^2 = 0$), so the prediction interval is numerically identical to the confidence interval; with 22 trials this estimate of τ^2 is itself imprecise, and the mortality prediction interval should be regarded as a lower

bound on the true predictive uncertainty. All three HVPG reductions lie within the range observed across the included trials.

Table S4: Sensitivity Analysis: Alternative Weighting Schemes

Comparison of meta-regression results across different weighting approaches. All odds ratios are expressed as protective ORs (OR < 1 indicates benefit with HVPG reduction) per 1 mmHg HVPG reduction.

Outcome	Weighting Scheme	OR per mmHg	95% CI	P-value	τ^2	I^2 (%)
**Mortality**	Outcome-based (standard)	0.850	0.771–0.937	0.004	0.000	0.0
	Predictor-based	0.810	0.687–0.955	0.021	0.131	21.7
	Joint weighting	0.857	0.775–0.947	0.007	0.000	0.0
	Iteratively reweighted	0.856	0.775–0.947	0.007	0.000	0.0
**Varices**	Outcome-based (standard)	0.852	0.744–0.976	0.032	0.071	18.8
	Predictor-based	0.734	0.594–0.908	0.010	0.530	52.9
	Joint weighting	0.849	0.740–0.974	0.030	0.061	15.4
	Iteratively reweighted	0.849	0.740–0.974	0.030	0.060	15.3
**Bleeding**	Outcome-based (standard)	0.871	0.741–1.024	0.111	0.050	7.3
	Predictor-based	0.743	0.593–0.931	0.018	0.632	57.2
	Joint weighting	0.868	0.736–1.023	0.106	0.037	5.2
	Iteratively reweighted	0.867	0.736–1.023	0.106	0.037	5.2

OR = odds ratio per 1 mmHg HVPG reduction (protective convention: OR < 1 = benefit). Outcome-based weighting is the primary analysis. τ^2 = between-study variance; I^2 = percentage of heterogeneity.

Table S5: Sensitivity to the assumed baseline/follow-up correlation (ρ), all outcomes and all weighting schemes

Meta-regression results under varying assumptions for the correlation between baseline and follow-up HVPG measurements (ρ), used to impute the SD of HVPG change. Because ρ affects only the variance of the predictor and not its point estimate, the outcome-weighted primary analysis is invariant to ρ by construction; ρ can influence only the predictor-weighted and joint schemes. Across ρ = 0.5-0.8 those schemes varied by less than 5% in the odds ratio per mmHg and never changed the direction of the association. Results are shown for all three outcomes.

A. All-cause mortality

ρ	Outcome-weighted (primary)	Predictor-weighted	Joint weighting
0.5	0.850 (0.771-0.937), p=0.004	0.792 (0.675-0.930), p=0.010	0.859 (0.777-0.950), p=0.008
0.6	0.850 (0.771-0.937), p=0.004	0.800 (0.680-0.942), p=0.014	0.858 (0.776-0.949), p=0.007
0.687 (primary)	0.850 (0.771-0.937), p=0.004	0.810 (0.687-0.955), p=0.021	0.857 (0.775-0.947), p=0.007
0.7	0.850 (0.771-0.937), p=0.004	0.812 (0.688-0.958), p=0.022	0.857 (0.775-0.947), p=0.007
0.8	0.850 (0.771-0.937), p=0.004	0.827 (0.698-0.979), p=0.039	0.855 (0.774-0.944), p=0.006

B. Variceal events

ρ	Outcome-weighted (primary)	Predictor-weighted	Joint weighting
0.5	0.852 (0.744-0.976), p=0.032	0.724 (0.588-0.891), p=0.006	0.848 (0.739-0.972), p=0.029
0.6	0.852 (0.744-0.976), p=0.032	0.729 (0.591-0.899), p=0.008	0.848 (0.740-0.973), p=0.029
0.687 (primary)	0.852 (0.744-0.976), p=0.032	0.734 (0.594-0.908), p=0.010	0.849 (0.740-0.974), p=0.030
0.7	0.852 (0.744-0.976), p=0.032	0.735 (0.595-0.909), p=0.010	0.849 (0.740-0.974), p=0.030
0.8	0.852 (0.744–0.976), p=0.032	0.744 (0.600-0.922), p=0.014	0.849 (0.740-0.974), p=0.030

C. Variceal bleeding

ρ	Outcome-weighted (primary)	Predictor-weighted	Joint weighting
0.5	0.871 (0.741-1.024), p=0.111	0.729 (0.585-0.908), p=0.011	0.866 (0.734-1.022), p=0.104
0.6	0.871 (0.741-1.024), p=0.111	0.736 (0.589-0.920), p=0.014	0.867 (0.735-1.022), p=0.105
0.687 (primary)	0.871 (0.741-1.024), p=0.111	0.743 (0.593-0.931), p=0.018	0.868 (0.736-1.023), p=0.106

ρ	Outcome-weighted (primary)	Predictor-weighted	Joint weighting
0.7	0.871 (0.741-1.024), p=0.111	0.744 (0.593-0.933), p=0.019	0.868 (0.736-1.023), p=0.106
0.8	0.871 (0.741-1.024), p=0.111	0.754 (0.599-0.949), p=0.026	0.869 (0.738-1.023), p=0.107

Odds ratio per 1 mmHg greater between-group HVPG reduction in the treatment arm (protective convention: OR < 1 = benefit). ρ = 0.687 is the data-derived median from the five trials reporting sufficient information to back-calculate it.

Table S6: Leave-One-Out Sensitivity Analysis

Results of meta-regression after sequential removal of each study. Odds ratios are expressed as protective ORs per 1 mmHg HVPG reduction. Results are shown for mortality and variceal events (the two outcomes reaching statistical significance in the primary analysis).

	Mortality			Varices		
**Study Excluded**	**OR**	**95% CI**	**P**	**OR**	**95% CI**	**P**
Ranjan 2024	0.850	0.772–0.937	0.004	0.851	0.745–0.973	0.029
Kronborg 2023	0.854	0.772–0.944	0.006	0.855	0.743–0.984	0.042
Gupta 2013	0.849	0.771–0.935	0.004	0.854	0.744–0.979	0.035
Vijayaraghaven 2020	0.845	0.767–0.931	0.003	0.855	0.743–0.984	0.041
Garcia-Tsao 2020	0.854	0.776–0.939	0.004	0.857	0.749–0.980	0.037
Kumar 2019	0.846	0.767–0.932	0.003	0.853	0.743–0.980	0.037
Burroughs 1983	0.847	0.766–0.936	0.004	0.841	0.735–0.963	0.022
Abecasis 2003	0.847	0.768–0.934	0.004	0.849	0.738–0.977	0.034
Villeneuve 1986	0.879	0.790–0.979	0.030	0.850	0.732–0.987	0.047
Merkel 2004	0.849	0.768–0.939	0.005	0.844	0.749–0.951	0.012
Sarin 2013	0.849	0.772–0.933	0.003	0.850	0.744–0.972	0.028
Bishnu 2018	0.850	0.770–0.939	0.005	0.851	0.741–0.978	0.034
Conn 1991	0.849	0.768–0.939	0.005	0.845	0.742–0.962	0.020
Groszmann 2005	0.844	0.763–0.933	0.004	**0.880**	**0.771–1.004**	**0.072**
Bhardwaj 2016	0.838	0.768–0.914	0.001	0.841	0.729–0.970	0.028
Villanueva 2019	0.849	0.769–0.938	0.004	0.851	0.739–0.980	0.037
Jenkins 1997	0.866	0.785–0.957	0.011	**0.868**	**0.750–1.005**	**0.073**
Hidaka 2011	0.850	0.769–0.939	0.005	0.852	0.742–0.979	0.036
Chalasani 2019	0.851	0.771–0.940	0.005	0.849	0.736–0.981	0.038
Harrison 2018	0.851	0.770–0.941	0.005	0.861	0.756–0.981	0.036
Tevethia 2024	0.853	0.766–0.950	0.009	0.845	0.727–0.981	0.040
Khajuria 2025	0.823	0.719–0.942	0.011	**0.842**	**0.691–1.025**	**0.103**
**Primary analysis (all studies)**	**0.850**	**0.771–0.937**	**0.004**	**0.852**	**0.744–0.976**	**0.032**

OR = protective odds ratio per 1 mmHg HVPG reduction. Bold values for variceal events indicate studies whose removal shifts the p-value above 0.05 (Groszmann 2005, Jenkins 1997, Khajuria 2025). All mortality results remained statistically significant regardless of which study was excluded.

Table S7. One-stage binomial-normal model versus the two-stage primary analysis

Outcome	Model	OR per mmHg (95% CI)	P
All-cause mortality	Two-stage (primary)	0.850 (0.771–0.937)	0.004
	One-stage (binomial-normal)	0.856 (0.745–0.983)	0.028
Variceal events	Two-stage (primary)	0.852 (0.744–0.976)	0.032
	One-stage (binomial-normal)	0.853 (0.749–0.971)	0.016
Variceal bleeding	Two-stage (primary)	0.871 (0.741–1.024)	0.111
	One-stage (binomial-normal)	0.857 (0.723–1.016)	0.076

Two-stage: random-effects meta-regression of study-level log odds ratios (REML, Knapp–Hartung), with a 0.5 continuity correction applied only to arms with zero events. One-stage: random-effects logistic (binomial-normal) meta-regression fitted to the event counts directly (unconditional model with random study intercepts), which requires no continuity correction. Trials with a zero-event arm: mortality: 5 trials (Garcia-Tsao, Bishnu, Jenkins, Hidaka, Chalasani, of which Hidaka is double-zero); variceal events: 2 (Garcia-Tsao, Harrison); bleeding: 5 (Garcia-Tsao, Bhardwaj, Hidaka, Chalasani, Harrison, of which Bhardwaj and Hidaka are double-zero).

Table S8. Influence diagnostics for each included trial

Studies are ordered by Cook's distance. No trial reached Cook's D > 1 for any outcome.

A. All-cause mortality (largest Cook's D = 0.682, Villeneuve 1986)

Study	Cook's D	DFBETAS (slope)	Studentized residual	Hat value	Covariance ratio
Villeneuve 1986	0.682	-0.703	+1.36	0.277	1.27
Khajuria 2025	0.482	+0.646	+0.69	0.497	2.10
Bhardwaj 2016	0.232	+0.332	-2.55	0.043	0.64
Kumar 2019	0.182	+0.104	+1.13	0.126	1.11
Jenkins 1997	0.166	-0.396	-1.37	0.084	1.00
Ranjan 2024	0.103	-0.004	-1.09	0.080	1.07
Groszmann 2005	0.075	+0.147	-0.68	0.135	1.22
Vijayaraghaven 2020	0.029	+0.111	-1.13	0.023	1.00
Villanueva 2019	0.026	+0.013	+0.55	0.076	1.16
Sarin 2013	0.025	+0.032	+1.51	0.012	0.89
Garcia-Tsao 2020	0.024	-0.091	+1.47	0.012	0.90
Kronborg 2023	0.020	-0.095	+0.54	0.061	1.15
Abecasis 2003	0.017	+0.065	-0.98	0.017	1.02
Gupta 2013	0.016	+0.023	-1.16	0.012	0.98
Burroughs 1983	0.014	+0.074	+0.50	0.053	1.14
Tevethia 2024	0.010	-0.075	-0.19	0.200	1.38
Merkel 2004	0.009	+0.017	-0.21	0.165	1.32
Chalasani 2019	0.004	-0.038	+0.61	0.010	1.08

Study	Cook's D	DFBETAS (slope)	Studentized residual	Hat value	Covariance ratio
Bishnu 2018	0.001	-0.010	-0.45	0.007	1.09
Harrison 2018	0.001	-0.024	+0.22	0.023	1.13
Conn 1991	0.001	+0.012	-0.10	0.082	1.21
Hidaka 2011	0.001	+0.004	+0.38	0.004	1.10

B. Variceal events (largest Cook's D = 0.566, Groszmann 2005)

Study	Cook's D	DFBETAS (slope)	Studentized residual	Hat value	Covariance ratio
Groszmann 2005	0.566	-0.495	+1.63	0.176	0.93
Merkel 2004	0.429	+0.153	-2.29	0.077	0.61
Burroughs 1983	0.106	+0.186	+1.27	0.065	1.00
Ranjan 2024	0.089	+0.011	-1.23	0.056	0.98
Conn 1991	0.083	+0.129	-1.68	0.032	0.85
Bhardwaj 2016	0.081	+0.178	-0.72	0.132	1.24
Jenkins 1997	0.079	-0.262	-0.75	0.122	1.20
Sarin 2013	0.065	+0.027	+1.34	0.037	0.95
Harrison 2018	0.044	-0.156	+1.72	0.016	0.84
Khajuria 2025	0.034	+0.166	+0.16	0.526	2.42
Kumar 2019	0.027	-0.023	-0.71	0.050	1.13
Tevethia 2024	0.026	+0.118	+0.29	0.193	1.43
Garcia-Tsao 2020	0.018	-0.080	+1.24	0.012	0.96
Villanueva 2019	0.018	+0.016	+0.39	0.092	1.26

Study	Cook's D	DFBETAS (slope)	Studentized residual	Hat value	Covariance ratio
Hidaka 2011	0.012	-0.004	-0.64	0.029	1.11
Gupta 2013	0.011	-0.024	+0.91	0.013	1.03
Abecasis 2003	0.010	+0.055	-0.47	0.042	1.15
Bishnu 2018	0.010	+0.020	+0.61	0.023	1.10
Kronborg 2023	0.007	-0.053	+0.44	0.031	1.13
Vijayaraghaven 2020	0.006	-0.046	+0.43	0.029	1.13
Chalasani 2019	0.004	+0.044	-0.17	0.095	1.28
Villeneuve 1986	0.001	+0.030	-0.06	0.152	1.36

C. Variceal bleeding (largest Cook's D = 0.281, Khajuria 2025)

Study	Cook's D	DFBETAS (slope)	Studentized residual	Hat value	Covariance ratio
Khajuria 2025	0.281	+0.494	+0.57	0.403	1.98
Ranjan 2024	0.248	+0.095	-1.85	0.105	0.80
Jenkins 1997	0.239	-0.451	-1.10	0.164	1.19
Conn 1991	0.173	+0.246	-2.04	0.062	0.74
Villeneuve 1986	0.129	+0.266	-0.38	0.288	1.67
Harrison 2018	0.114	-0.249	+1.64	0.030	0.82
Merkel 2004	0.113	+0.125	-1.56	0.051	0.91
Burroughs 1983	0.099	+0.123	+0.94	0.097	1.11
Abecasis 2003	0.092	+0.173	-0.75	0.082	1.22
Villanueva 2019	0.079	-0.066	+1.08	0.048	0.99

Study	Cook's D	DFBETAS (slope)	Studentized residual	Hat value	Covariance ratio
Kumar 2019	0.073	-0.020	+0.95	0.064	1.05
Tevethia 2024	0.067	-0.015	-0.29	0.285	1.65
Chalasani 2019	0.053	-0.153	+1.27	0.024	0.93
Garcia-Tsao 2020	0.042	-0.131	+1.15	0.023	0.96
Sarin 2013	0.023	-0.018	+0.99	0.019	1.00
Gupta 2013	0.017	-0.048	+0.77	0.025	1.06
Groszmann 2005	0.008	+0.051	-0.13	0.055	1.23
Bishnu 2018	0.003	+0.024	+0.39	0.039	1.18
Kronborg 2023	0.003	-0.040	+0.30	0.059	1.20
Vijayaraghaven 2020	0.002	-0.030	+0.28	0.055	1.20
Hidaka 2011	0.000	+0.002	+0.26	0.008	1.12
Bhardwaj 2016	0.000	-0.007	+0.15	0.014	1.13

Cook's distance quantifies the overall change in the fitted meta-regression when a trial is removed; DFBETAS is the standardised change in the slope; the hat value is the leverage of a trial in the predictor; a covariance ratio above 1 indicates a trial that increases the precision of the estimates when retained. Computed with metafor::cooks.distance.rma.uni and metafor::influence.rma.uni.

Table S9. Measurement-error correction: SIMEX-disattenuated slopes

Outcome	Assumed source of ΔHVPG measurement error	Naive OR	Disattenuated OR	95% CI	Attenuation
All-cause mortality	Trial-specific sampling variance of ΔHVPG (primary)	0.850	0.823	(0.676–0.921)	20.1%
	External test–retest SD 2.19 mmHg (compensated; Bai 2021)	0.850	0.835	—	11.0%
	External test–retest SD 2.63 mmHg (decompensated; Bai 2021)	0.850	0.828	—	16.1%
Variceal events	Trial-specific sampling variance of ΔHVPG (primary)	0.852	0.832	(0.665–0.955)	14.6%
	External test–retest SD 2.19 mmHg (compensated; Bai 2021)	0.852	0.840	—	9.1%
	External test–retest SD 2.63 mmHg (decompensated; Bai 2021)	0.852	0.838	—	10.5%
Variceal bleeding	Trial-specific sampling variance of ΔHVPG (primary)	0.871	0.854	(0.677–0.972)	14.1%
	External test–retest SD 2.19 mmHg (compensated; Bai 2021)	0.871	0.859	—	9.9%
	External test–retest SD 2.63 mmHg (decompensated; Bai 2021)	0.871	0.862	—	7.6%

Odds ratio per 1 mmHg greater between-group HVPG reduction, before and after SIMEX correction for measurement error in the predictor. The primary correction uses each trial own sampling variance of the between-group ΔHVPG, which is the quantity that attenuates a trial-level slope and requires no external test-retest data; its 95% CI is from a study-level bootstrap. The two external scenarios substitute the published test-retest SD of ΔHVPG in untreated patients from Bai et al. [2] (Table 2, "Change SD": 2.19 mmHg in compensated and 2.63 mmHg

in decompensated cirrhosis) and are presented as bounding scenarios only, without bootstrap intervals. Attenuation is the proportion of the log-odds slope recovered by the correction.

Supplementary references

1. Sterne JAC, Savović J, Page MJ, et al. RoB 2: a revised tool for assessing risk of bias in randomised trials. BMJ 2019;366:l4898.

2. Bai W, Al-Karaghouli M, Stach J, et al. Test-Retest Reliability and Consistency of HVPG and Impact on Trial Design: A Study in 289 Patients from 20 Randomized Controlled Trials. Hepatology 2021;74:3301-3315.
