# Supplementary material for: Magnitude of portal pressure reduction and clinical outcomes in randomized trials
Source: JHEP Rep. 2026 Sep 21;8(12):102028. doi: 10.1016/j.jhepr.2026.102028 (PMC13756290; doi:10.1016/j.jhepr.2026.102028)
Supplement: Supplementary file 3 [file mmc2.docx]

**Supplementary CTAT Table**

*Tables for a "Complete, Transparent, Accurate and Timely account" (CTAT)*

Manuscript JHEPR-D-26-01048 — "Magnitude of HVPG Reduction and Clinical Outcomes in Portal Hypertension: Systematic Review Meta-Regression Analysis of RCTs"

This study is a systematic review and meta-regression of previously published randomised controlled trials. No new human or animal subjects, biological samples, antibodies, cell lines, organisms or sequence-based reagents were used, so sections 1.1–1.5 and 1.8 of the CTAT template are not applicable and have been omitted in line with the instruction to include only the parts relevant to the study.

**1.6 Deposited data**

| **Name of repository** | **Identifier** | **Link** |
| --- | --- | --- |
| Extracted trial-level dataset (22 RCTs) and full analysis code | Available in the paper from tables | — |
| Full analysis code | Available from authors upon reasonable request |  |

**1.7 Software**

| **Software name** | **Manufacturer** | **Version** |
| --- | --- | --- |
| R | R Foundation for Statistical Computing, Vienna, Austria | 4.5.0 |
| metafor | W. Viechtbauer (CRAN) | 4.8.0 |
| mvmeta | A. Gasparrini (CRAN) | 1.0.3 |
| rms | F.E. Harrell Jr (CRAN) | 8.0.0 |
| tidyverse | Posit PBC (CRAN) | 2.0.0 |
| ggplot2 | Posit PBC (CRAN) | 3.5.2 |
| patchwork | T.L. Pedersen (CRAN) | 1.3.0 |
| scales | Posit PBC (CRAN) | 1.4.0 |
| knitr | Y. Xie (CRAN) | 1.5 |
| rmarkdown | Posit PBC (CRAN) | 2.29 |
| Covidence | Veritas Health Innovation, Melbourne, Australia | Web application, accessed 2020–2025 |

**1.9 Corresponding methods author(s)**

| **Name** | **Affiliation** | **e-mail** |
| --- | --- | --- |
| Juan G. Abraldes | Liver Unit, Division of Gastroenterology, Department of Medicine, University of Alberta, Edmonton, Canada | juan.g.abraldes@ualberta.ca |

**2.0 Clinical protocol confirmation**

Not applicable — this study is a systematic review and meta-regression of published randomised controlled trials and did not itself enrol participants. The review protocol, including eligibility criteria, outcomes and the analysis plan, was defined before data extraction but was not prospectively registered and no public protocol was posted. Reporting follows PRISMA 2020 (checklist provided as Supplementary Material). Screening and data extraction were performed independently by two reviewers with third-reviewer adjudication.
